# Atropia in Epilepsy

**Published:** 1878-08

**Authors:** 


Atropia in Epilepsy.—A trial has been made in Dr. Leidesdorf's clinic of the continued use of *small* doses of atropia in cases of epilepsy. It was found to act beneficially, if given to the extent of one-seventieth grain of the sulphate daily. The trial was made in consequence of the accepted fact that small doses diminish the action of the reflex nerve centres, while large doses produce an opposite effect. Cases of motor epilepsy of not long standing recovered rapidly, and some old cases complicated with mental derangement also got well while taking the drug. In other cases the attacks were rendered less frequent. The observation was confirmed by experiments on animals.—*London Medical Examiner.*

